# Helium Plasma-Assisted Breast Reduction: A Pilot Study

**DOI:** 10.1093/asjof/ojac041

**Published:** 2022-05-06

**Authors:** Seraphine Moncada, Christopher Nichols

## Abstract

**Background:**

Reduction mammaplasty and mastopexy are currently some of the most performed breast procedures. Techniques typically involve deepithelialization of the nipple-areola complex pedicle. Traditionally, scalpel or scissor dissection is performed below the basal skin layer to remove the germinal epithelium but above the subcutis to preserve the subdermal vascular plexus. Deepithelialization thus leaves a strong dermal “leash” for the pedicle while preserving the subdermal blood supply. This process is time intensive and bloody, and often an assistant is required for countertraction. Previously, authors have described laser-assisted breast reduction surgery as an alternative to traditional cold knife techniques. The advent of helium plasma generators offers another option for deepithelialization. This study is a preliminary assessment of the safety and efficacy of this application.

**Objectives:**

The authors performed a prospective pilot study of 10 patients who underwent outpatient, inferior pedicle, breast reduction mammaplasty, or mastopexy surgery by a single surgeon. Outcomes were assessed for safety and efficacy. Representative tissue samples were evaluated by an independent pathology group.

**Methods:**

All patients received standard outpatient perioperative care. Deepithelialization was performed using the Renuvion helium plasma device (Apyx Medical, Clearwater, FL), and standard breast reduction or mastopexy was performed.

**Results:**

No major complications occurred in our series. Minor complications occurred in 1 patient (10%). No inclusion cysts were recorded in any patients.

**Conclusions:**

Helium plasma energy for deepithelialization in breast reduction was found to be safe, efficient, and effective. Decreased operating room time and blood loss suggest that helium plasma is a potential alternative for surgeons who have access to this technology.

**Level of Evidence: 5:**

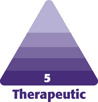

This preliminary study was designed to investigate the efficacy of helium plasma-based pedicle deepithelialization for use in breast surgery. Specifically, we examined the safety and efficacy of this energy modality in mastopexy and reduction mammaplasty.

Deepithelialization was first described by Dr Schwartzman in 1937 and is a common tenet of mammaplasty in techniques that use dermal and dermoglandular pedicles to support the repositioning of the nipple-areola complex and parenchyma.^1^ This portion of the procedure is typically time consuming and labor intensive, especially when the breasts are large and pendulous and/or when there is no qualified assistant available for retraction. The epithelial layer of the skin must be painstakingly removed with a scalpel or scissors and often requires an assistant for countertraction and a breast tourniquet to keep proper skin tension to develop and maintain a consistent depth of dissection and preserve the integrity of the subdermal vasculature plexus.

Existing studies have shown the effectiveness of laser devices^2-5^ for this application, and this has become a well-established technique.^6,7^ However, laser generators are very costly, require special eye and airway protection for the surgical team and the patient, and are cumbersome in the operating room (OR) as they are not specifically designed to be used intraoperatively or within a sterile field. In this study, the authors seek to expand upon the existing techniques by examining similar surgical maneuvers with a helium plasma device rather than a laser. Although the method of delivering the energy required for deepithelialization of the skin differs (helium plasma vs carbon dioxide laser), the amount of energy delivered is identical (120 W), and the hypothesis is that the surgical effect and endpoint are the same. Additionally, the plasma generator and handpiece are primarily designed for and well adapted to use in the intraoperative surgical field.

## METHODS

The study was designed and Sterling IRB approval for the study was obtained. All patients presenting to the practice of the senior author (C.N.) during the accrual period were given the opportunity to participate in the study until 10 study patients were accrued. The study goals, endpoints, and risks were reviewed with the patient as well as the standard risks, benefits, and alternatives of the proposed procedure. Informed consent for the surgical procedure as well as for participation in the study and the accrual of study data was obtained. The study range was from September 2021 to November 2021.

On the day of surgery, patients were measured and marked. An appropriate weight-based dose of preoperative antibiotics was administered. All patients were treated with lower extremity sequential compression devices before the induction of general anesthesia. Betadine or chlorhexidine skin prep was utilized, and the breasts were pretreated by infiltration with a solution of 0.4 mg lidocaine/cc and 1:1,000,000 epinephrine. This mixture was injected into the planned regions of dissection in the superior, medial, and lateral breast.

Marked skin incisions were made. The Renuvion/Jplasma generator (Apyx Medical, Clearwater, FL) with a BVX-044-BPS J-Plasma Precise Open 44-mm handpiece was accessed. Settings were placed at 120 W, 4 liters helium flow using the Cool-Coag energy setting. The plasma beam was utilized to precisely remove the epithelium from the skin of the previously marked dermoglandular pedicle. Care was taken to uniformly “paint” the tissue with overlapping strokes to avoid any skips. No assistant or tourniquet for countertraction was required for this portion of the procedure. The eschar of carbonized epithelial debris from the treatment was then removed by wiping with a saline moistened laparotomy sponge. A cuff of untreated skin around the plasma deepithelialized pedicle was excised and then a breast reduction or mastopexy was performed in the standard fashion. Drains were not used in the breasts. Patients were dressed with steri-strips, gauze, and a supportive bra. They were recovered in the surgery center and were discharged to home with instructions to ambulate the night of surgery.

Representative samples of the treated skin were sent for histopathologic evaluation. The specimen was formalin-fixed and paraffin embedded, and 4 µm sections were cut onto microscopic slides. Slides were stained with a Hematoxylin and eosin stain, cover slipped, and delivered to a pathologist for review.

## RESULTS

**Figure 1.**
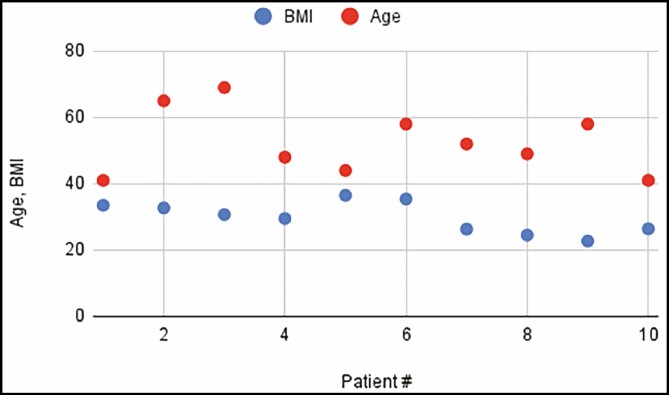
BMI and ages of patients.

**Figure 2.**
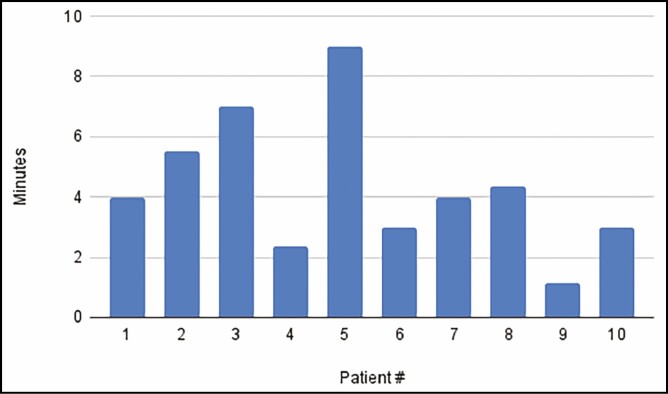
Average deepithelialization time (minutes) per patient, of left and right breasts.

**Figure 3.**
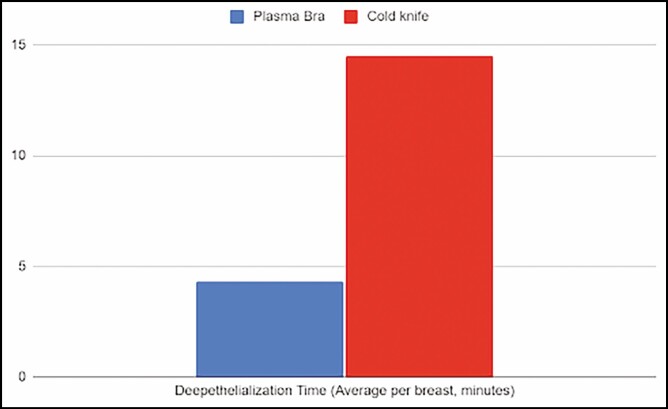
Deepithelialization time: average of plasma bra technique vs cold knife.

**Figure 4.**
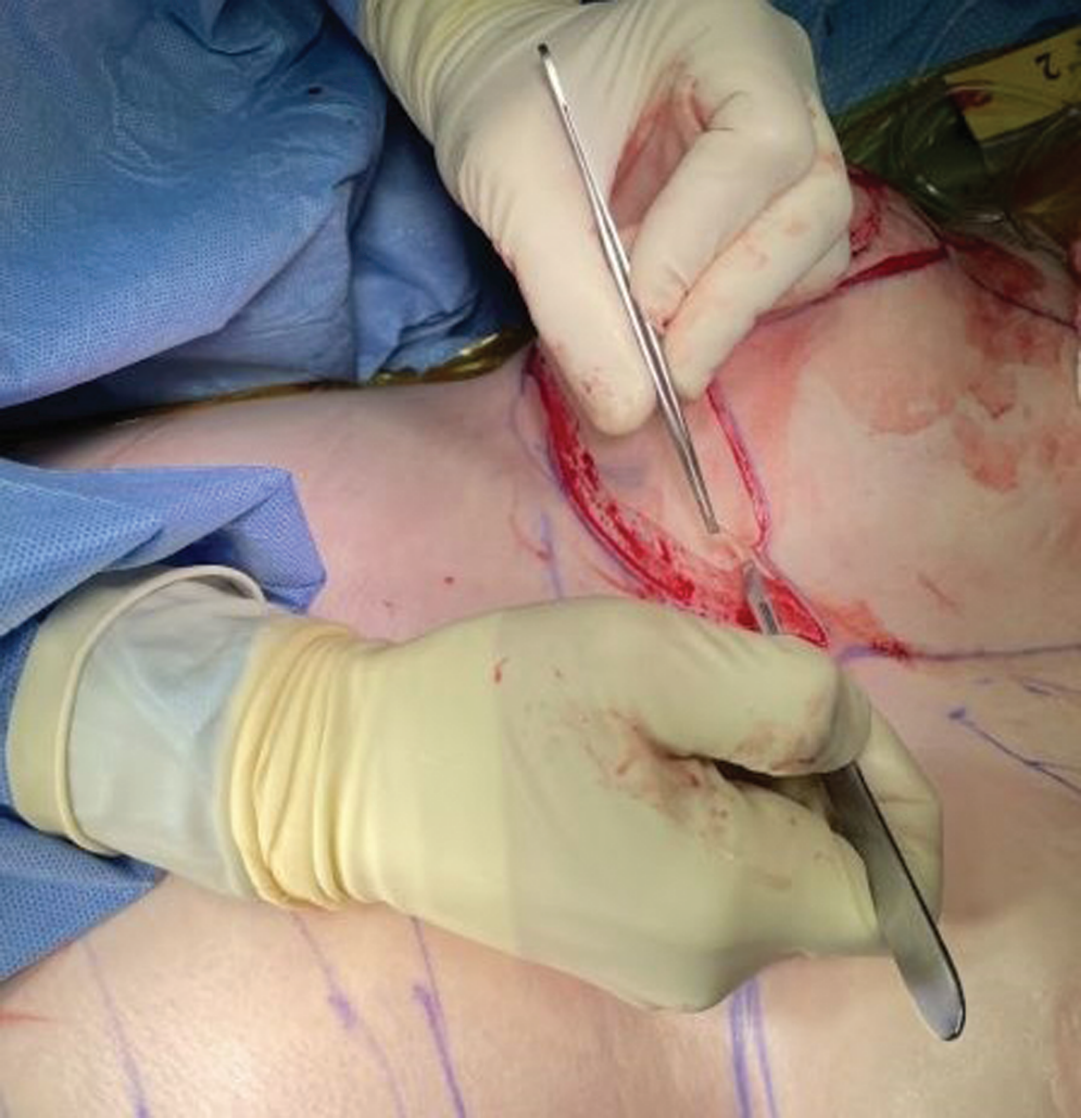
Cold knife deepithelialization technique.

**Figure 5.**
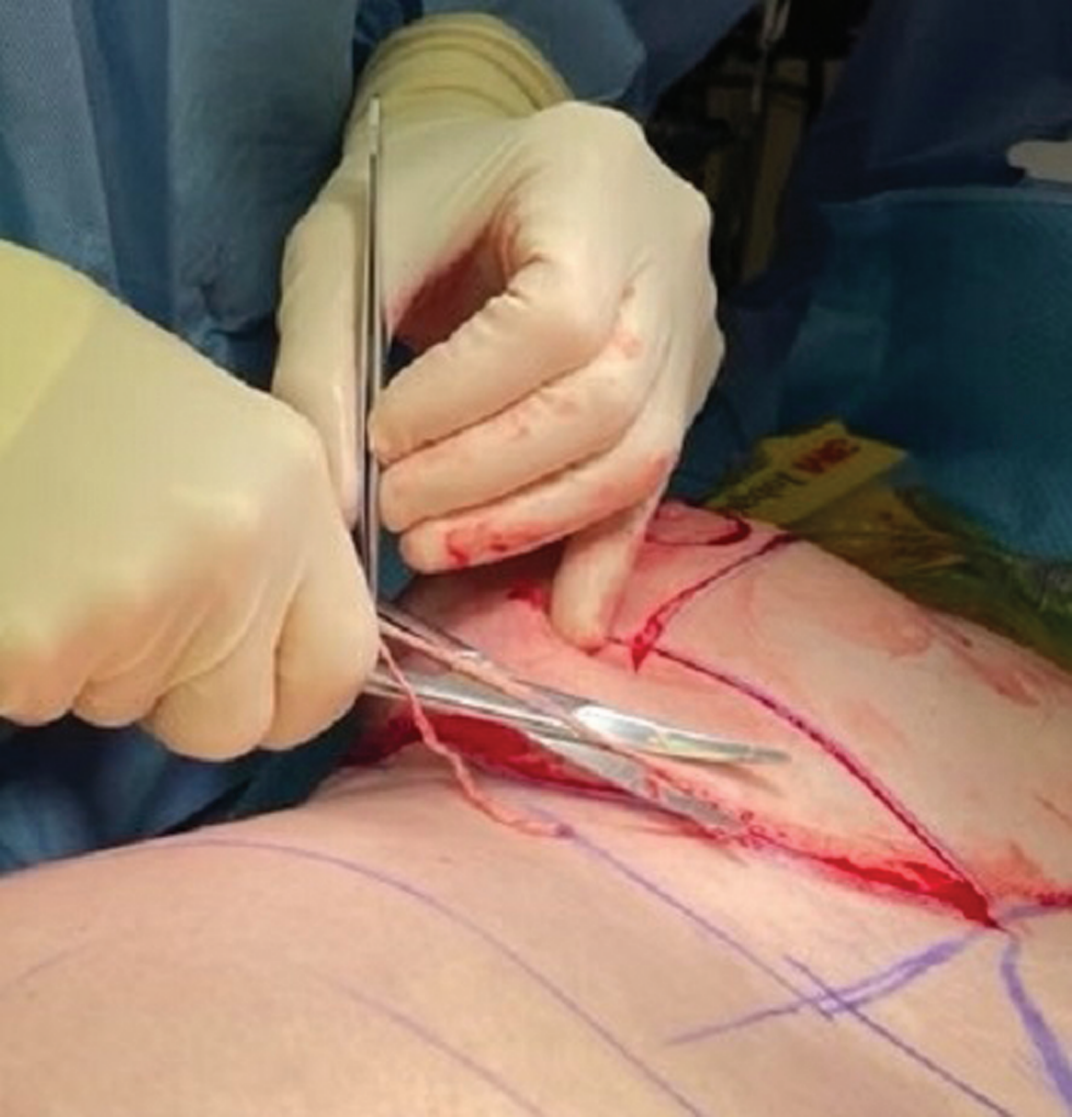
Scissor deepithelialization technique.

**Figure 6.**
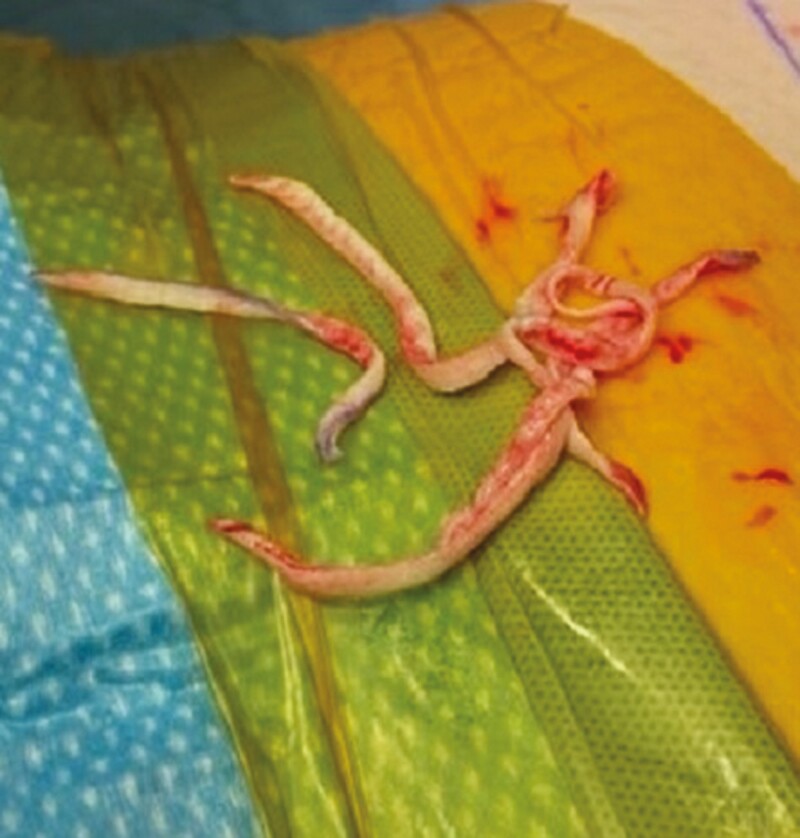
Skin remnants removed from the pedicle.

**Figure 7.**
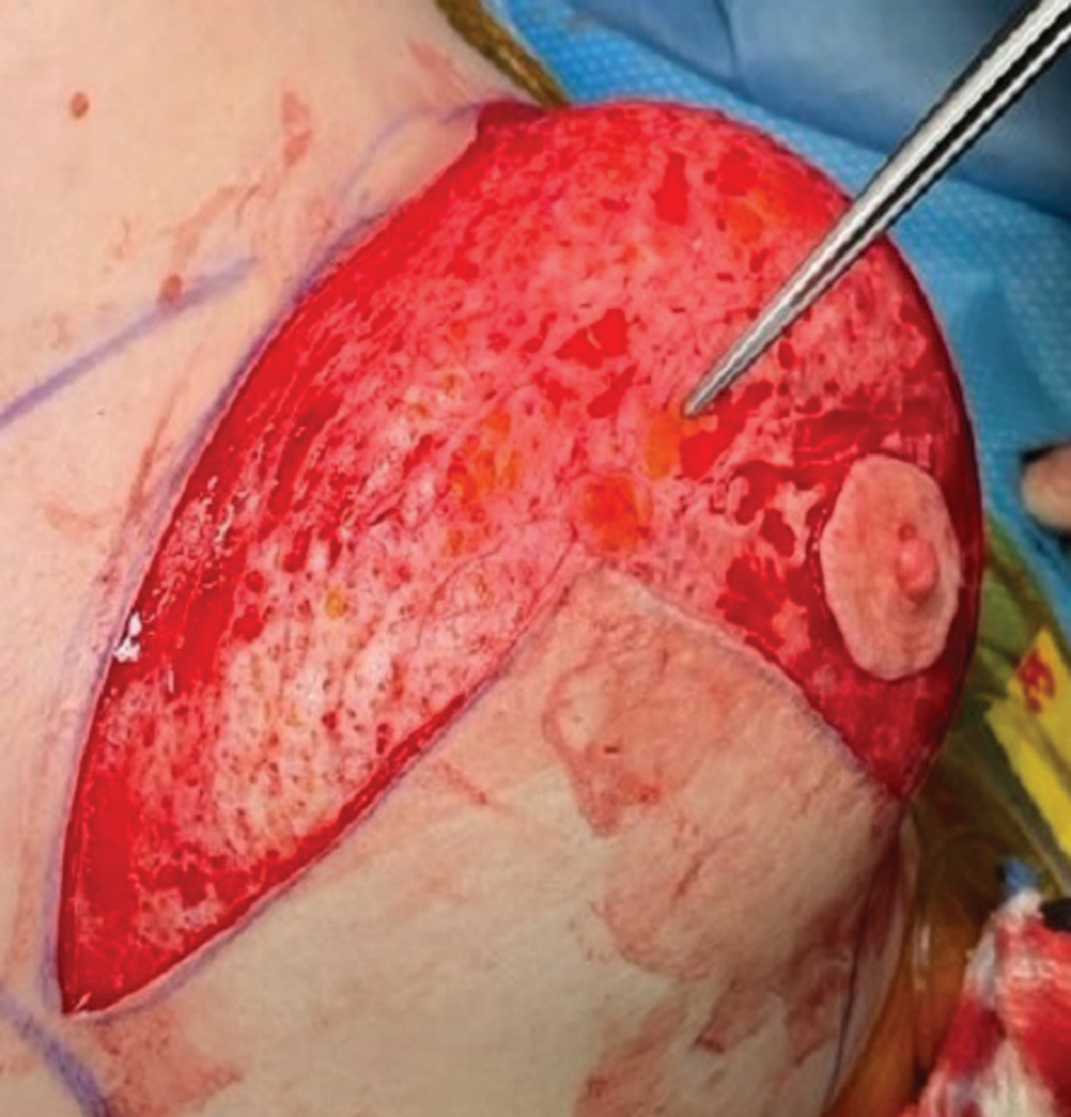
Irregularities and button holes in the dermis of the pedicle after cold knife deepithelialization.

**Figure 8.**
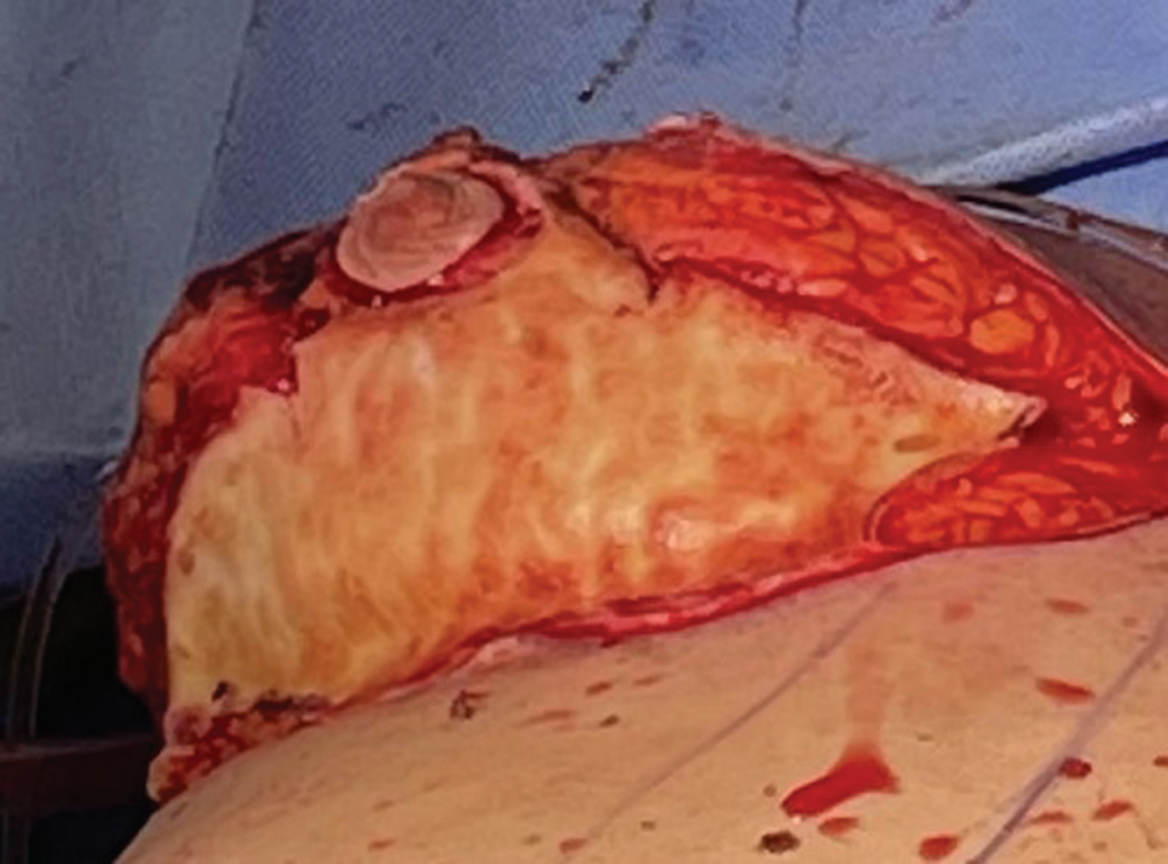
Robust, uniform dermal layer after plasma deepithelialization.

**Figure 9.**
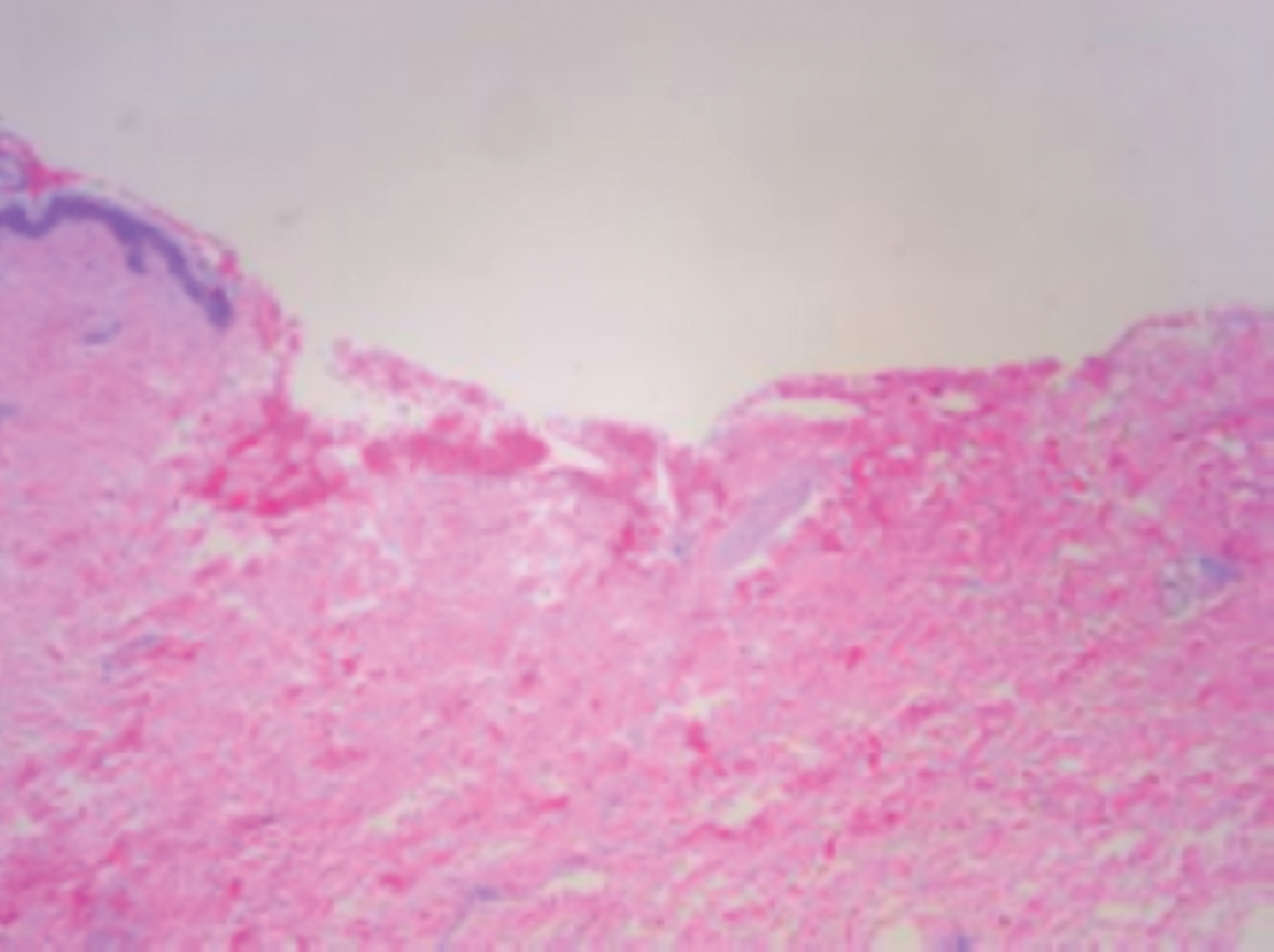
Cauterized skin absent of epidermis, with deep dermal vessels intact.

Ten patients were accrued into the study and underwent bilateral helium plasma-assisted, inferior pedicle, Wise pattern breast reduction or mastopexy. Patient ages ranged from 41 to 69 years old, with an average of 52.5 years. Patient BMI ranged from 22.7 to 35.4 kg/m² (Figure 1). Breast specimen weight ranged from skin only to 470 g. The average time for deepithelialization of the inferior pedicle was 4.2 minutes, ranging from 2 to 9 minutes per breast (Figure 2). Representative cold knife times for the same surgeon (C.N.) during the same period were 7-15 minutes per breast (Figure 3). There was no bleeding from the skin during this portion of the procedure, and the dermal leash and integrity of the pedicle were well maintained and significantly more uniform in contrast to the standard cold knife or scissor technique (Figures 4-8). In addition, significant contraction and shortening of the dermal length of the pedicle were observed. The average decrease in surface area of the treated region was 33.5%. Independent pathology evaluation for 3 representative samples showed skin with completely cauterized absence of epidermis with a depth of dermal damage at 0.5-, 1.0-, and 1.5-mm deep. Deep dermal vasculature and subcutis vasculature were intact. (Figure 9). The average follow-up time and range were 3 days postoperation to 6 months postoperation, with an average follow-up time of 6 weeks. 

No major complications were noted in our series. Minor complications occurred in 1 patient (10%), including 1 T-junction incisional wound breakdown, 0 infections, 0 hematomas, and 0 seromas. No inclusion cysts were recorded in any patients.

## DISCUSSION

This study demonstrates that helium plasma is a useful tool for deepithelialization of skin flaps. Some benefits of this technique include reliable preservation of the subdermal vascular plexus. It is fast, tidy, no remnant skin fragments on the field, bloodless, and precise with no potential for button holes into the subdermal fat. There is no need for countertraction from an assistant or breast tourniquet to render the skin taut for epidermal removal; thus, deepithelialization can easily be performed alone and at any time during the procedure, even after the pedicle and flaps have been elevated. In addition, due to the immediate tissue contraction, the pedicle is less unwieldy, and it contains stronger, more robust dermal tissue (similar in feel and suturing to a commercial acellular dermal matrix) to bolster parenchymal shaping sutures, especially in thinner or crepey skin (such as in weight loss patients). More long-term data will be required to determine if this tissue contraction has a durable effect in enhancing breast shape, but immediate on-table and early postoperative results show an impressive change. A drawback of this technique is that it relies on the availability of high-cost specialized equipment and disposables. A smoke plume is generated, which requires proper smoke evacuation equipment. Additionally, with the current design of the equipment, a resident or assistant surgeon cannot work simultaneously, unless 2 plasma generators are available.

This pilot study had some design limitations: The study was performed in an outpatient surgery center; therefore, patients with comorbidity or BMI parameters that would require hospital admission for surgery were not evaluated in this study. On March 14, 2022, the FDA published a safety communication related to the use of the Renuvion/J-Plasma device by Apyx Medical for certain aesthetic procedures intended to improve the appearance of the skin through dermal resurfacing or procedures under the skin for the purpose of skin tightening. The Renuvion/J-Plasma device used in this study has received clearance from the FDA for the cutting, coagulation, and ablation of soft tissue in open surgical procedures. The plasma bra procedure is an open surgical procedure involving the ablation of soft tissue. This use is, therefore, within the FDA clearance for the device and not one of the uses described in the FDA safety communication. Our experience has demonstrated that the Renuvion/J-Plasma device is both safe and effective for this application.

Laser energy has also been successfully utilized in this application. While both modalities are equally effective in thermally ablating the tissue, the helium plasma device and handpiece are specifically designed for sterile intraoperative use as a surgical instrument and may offer some ergonomic advantages. Additionally, no special laser precautions, drapes, protective equipment, eyewear, airway protection, etc. are required.

## CONCLUSIONS

**Figure 10.**
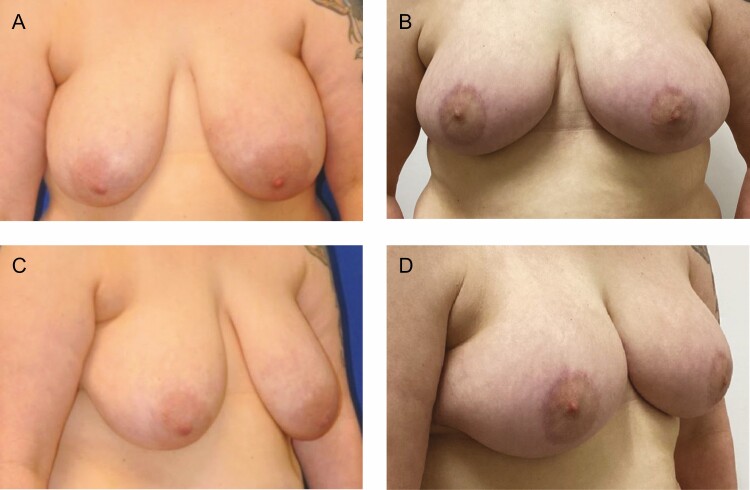
(A, C) Preoperative views of a 42-year-old female patient. (B, D) Postoperative views at 3 months from bilateral mastopexy using plasma bra technique.

**Figure 11.**
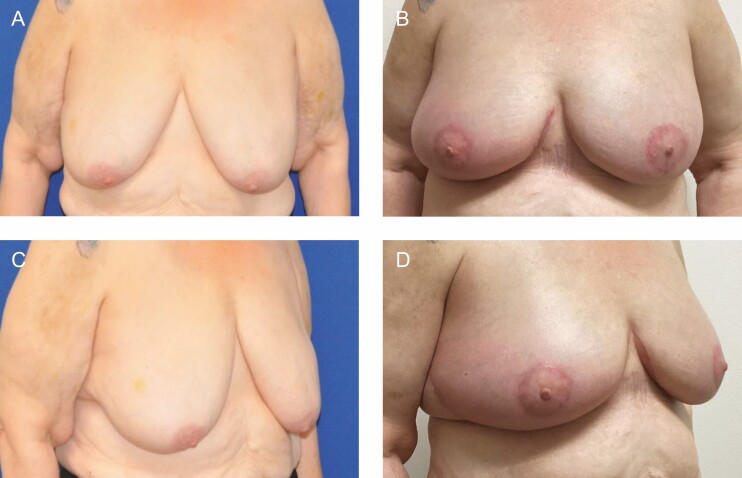
(A, C) Preoperative views of a 69-year-old female patient. (B, D) Postoperative views at 3 months from bilateral mastopexy using plasma bra technique.

**Figure 12.**
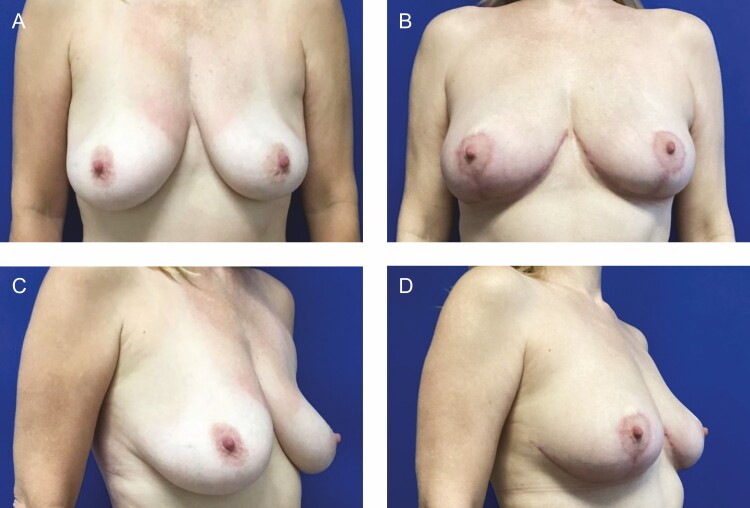
(A, C) Preoperative views of a 49-year-old female patient. (B, D) Postoperative views at 3 months from bilateral mastopexy using plasma bra technique.

This is the first report of the use of helium plasma energy in deepethelialization, which is well within the FDA indications for the device. The device limitations are for the delivery of radiofrequency and/or helium plasma for cutting, coagulation, and ablation of soft tissues during open surgical procedures.^6^ Dr Stevens’ group demonstrated excellent safety, efficacy, and results in a large series of patients and went on to popularize the “Laser Bra” procedure.^3^ The more recent widespread adoption of helium plasma energy-based surgery as an adjunct to liposuction has led to the increased availability of these devices in the OR. The study demonstrates that helium plasma deepithelialization is a safe, efficient, and precise alternative for use in breast reduction mammaplasty and mastopexy procedures. Benefits of this technique include a fast and bloodless dissection that does not require an assistant. Early observations and the obvious on-table thermal effect (Video) suggest a lifting/tightening effect on the tissue, which may potentially enhance the longevity of the new breast shape and aesthetic result (Figures 10-12). This preliminary study lacks the numerical power and length of follow-up to draw conclusions pertaining to improved quality of tissue lifting or the long-term effects on recurrent ptosis, but our preliminary observations are encouraging. Additionally, our complication rate compared favorably with published rates for mastopexy and breast reduction.^8-10^ Further study is ongoing to specifically assess these endpoints.

## Notes

Presented at: Renuvion Users’ Meeting in March 2022, Las Vegas, NV, USA.
